# Patient-Derived Xenograft Models for Endometrial Cancer Research

**DOI:** 10.3390/ijms19082431

**Published:** 2018-08-17

**Authors:** Cristian P. Moiola, Carlos Lopez-Gil, Silvia Cabrera, Angel Garcia, Tom Van Nyen, Daniela Annibali, Tina Fonnes, August Vidal, Alberto Villanueva, Xavier Matias-Guiu, Camilla Krakstad, Frédéric Amant, Antonio Gil-Moreno, Eva Colas

**Affiliations:** 1 Pathological Oncology Group, Biomedical Research Institute of Lleida (IRBLLEIDA), University Hospital Arnau de Vilanova, 25198 Lleida, Spain; 2 Biomedical Research Group in Gynecology, Vall Hebron Institute of Research, CIBERONC, 08035 Barcelona, Spain; carlos.lopez@vhir.org (C.L.-G.); agil@vhebron.net (A.G.-M.); 3 Gynecological Oncology Department, Vall Hebron University Hospital, 08035 Barcelona, Spain; scabrera@vhebron.net; 4 Pathology Department, Vall Hebron University Hospital, 08035 Barcelona, Spain; angarcia@vhebron.net; 5 Department of Oncology, Gynecological Oncology, KU Leuven, 3000 Leuven, Belgium; tom.vannyen@kuleuven.be (T.V.N.); daniela.annibali@kuleuven.be (D.A.); 6 Department of Obstetrics and Gynecology, Haukeland University Hospital, 5021 Bergen, Norway; Tina.Fonnes@uib.no (T.F.); camilla.krakstad@med.uib.no (C.K.); 7 Department of Pathology, University Hospital of Bellvitge, Oncobell, IDIBELL, CIBERONC, L’Hospitalet del Llobregat, 08907 Barcelona, Spain; avidal@bellvitgehospital.cat; 8 Xenopat S.L, Business Bioincubator, Bellvitge Health Science Campus, L’Hospitalet de Llobregat, 08907 Barcelona, Spain; avillanueva@iconcologia.net; 9 Chemoresistance and Predictive Factors Laboratory, ProCURE, Catalan Institute of Oncology (ICO), Oncobell, IDIBELL, L’Hospitalet de Llobregat, 08908 Barcelona, Spain; 10 Pathological Oncology Group and Department of Pathology, University Hospital Arnau de Vilanova, University of Lleida, 25198 Lleida, Spain; fjmatiasguiu.lleida.ics@gencat.cat; 11 University Hospital Bellvitge, IRBLLEIDA, IDIBELL, CIBERONC, 08907 Barcelona, Spain; 12 Centre for Gynecologic Oncology Amsterdam (CGOA), Antoni Van Leeuwenhoek-Netherlands Cancer Institute (AvL-NKI) and University Medical Centra (UMC), 1066 CX Amsterdam, The Netherlands; frederic.amant@uzleuven.be

**Keywords:** orthoxenograft, uterine cancer, avatar, murine models, personalized medicine, targeted therapy, preclinical studies, translational research

## Abstract

Endometrial cancer (EC) is the most common malignancy of the genital tract among women in developed countries. Recently, a molecular classification of EC has been performed providing a system that, in conjunction with histological observations, reliably improves EC classification and enhances patient management. Patient-derived xenograft models (PDX) represent nowadays a promising tool for translational research, since they closely resemble patient tumour features and retain molecular and histological features. In EC, PDX models have already been used, mainly as an individualized approach to evaluate the efficacy of novel therapies and to identify treatment-response biomarkers; however, their uses in more global or holistic approaches are still missing. As a collaborative effort within the ENITEC network, here we describe one of the most extensive EC PDX cohorts developed from primary tumour and metastasis covering all EC subtypes. Our models are histologically and molecularly characterized and represent an excellent reservoir of EC tumour samples for translational research. This review compiles the information on current methods of EC PDX generation and their utility and provides new perspectives for the exploitation of these valuable tools in order to increase the success ratio for translating results to clinical practice.

## 1. Introduction

### 1.1. Cancer Models

Establishing suitable models is one of the cornerstones for cancer research. Among the most important and challenging are mouse models, since they have to mimic different steps of the disease and are used as tools for biomarker identification as well as preclinical models for therapy drug screening [1,2]. In the last 60 years, murine cancer models have evolved from cell-line-derived to genetically engineered mice (GEM) and tissue allo- or xeno-graft models [3,4]. The main differences among these models are the genetic similarity between tumours and the host (isogenic, allogenic, or xenogenic); the site of tumour injection, implantation, growth, and development (heterotopic versus orthotopic); and the immunological status or immunocompetence of the host. Although cell-line-derived and GEM models have led to significant advances in cancer biology and are still crucial for cancer research [5], they often fail to recapitulate key aspects of human malignancies and thus do not adequately predict drug effects in the clinic. In fact, the high failure rate of preclinical compounds in clinical trials clearly demonstrates the limitations of existing preclinical models [6,7]. Thus, there is an urgent need to develop more realistic and clinically relevant mice models that reliably represent the patient`s tumour according to its genetic and molecular profile, its histopathology, the disease course and metastatic progression profile, and the therapy response [8]. The satisfaction of all these criteria will result in models with a close resemblance to human disease, enabling their use in preclinical trials with a high predictive value and significance for transferring results into the clinic. In this context, patient-derived xenograft (PDX) models have emerged as an excellent alternative to overcome these shortcomings [9,10]. PDX development is based on transplanting fresh cancer patient tissue samples directly into immunocompromised mice. In short, tumour tissue obtained directly from the operating room or from a biopsy is sliced into small fragments or disaggregated into cell suspension and surgically implanted or inoculated into immunocompromised mice. The most common mice strains used are SCID, NOD/SCID, NSG, and athymic nude mice [11]. The implantation or injection of the tumour fragment could be performed heterotopically or orthotopically. Tumours typically engraft over the course of weeks to months, depending mainly on tumour features (stage, grade, and aggressiveness). Upon engraftment and during exponential growth, the tumour is harvested and prepared for transplantation into one or various animals to develop a mice cohort of PDX that could be used for molecular characterization, biobanking, or as preclinical models. Serial expansions can take place for several passages maintaining tumour genetic fidelity [10,12].

As we have mentioned before, some research prefers to use tumour tissue fragments for PDX development, while other research uses a tumour cell suspension as the starting material. Each method has its advantages and disadvantages; tumour fragments retain cell–cell interactions as well as conserve tissue architecture, therefore mimicking better the tumour microenvironment. Alternatively, a single-cell suspension is a more heterogeneous sample that would represent unbiasedly the whole tumour; however, to obtain this type of sample, it is necessary to chemically or mechanically process the tissue, which affects cell viability and has the risk of decreasing engraftment success [13]. Also, the tumour implantation site is a crucial issue for PDX development. Heterotopic implantation occurs when the tumour fragment is implanted into an area of the mouse unrelated to the original tumour site, generally subcutaneously, in the interescapular region, the mammary fat pad, or the sub-renal capsular site. In contrast, orthotopical transplantation refers to when the patient’s tumour tissue is put into the corresponding anatomical organ as the original primary tumour. Subcutaneous PDX rarely develop metastases in mice and it is difficult to use them to simulate the initial tumour microenvironment. In contrast, orthotopic models can mimic the natural environment of a primary tumour, turning it into an ideal model to study the metastatic process. Nevertheless, these models can be difficult to generate depending on the organ of implantation [14].

In summary, the main features and advantages of PDX models are: (1) preservation of the genetic profile of the primary tumour and stability along passages into several animals; (2) retention of the histological and phenotypic features of the tumour, such as its tissue architecture, and the maintenance of stromal and stem cell components, cell-to-cell interactions, and spatial distribution; (3) amplification of tumour tissue biomass to facilitate tumour biology analysis, such as tumour characterization and biomarker identification; and (4) generation of a mice cohort with the same tumour that can be used as a preclinical model to test and predict anticancer drug response [15].

### 1.2. Endometrial Cancer

Endometrial cancer (EC) is the most common gynaecological cancer in developed countries and the sixth in mortality among all cancer types in women [16]. The majority of ECs are diagnosed at early stages when the tumour is still confined to the uterus and are frequently associated with vaginal bleeding as an initial symptom [17,18,19]. EC early detection is crucial to increase patient survival: the 5-year overall survival rate is around 80–95% in early stage tumours and falls to 20–60% in more advanced tumours [20,21].

Classically, EC has been classified into a dualistic model according to its biological, molecular, and clinical features. Type I or endometrioid endometrial carcinomas, comprising 80% of cases, are mainly represented by low-grade and hormone-receptor-positive tumours, while type II or non-endometrioid endometrial carcinomas are represented by papillary serous carcinoma, clear cell carcinoma, and carcinosarcoma, among other minor histologies, and are characterized by high-grade tumours and loss of hormone receptors [22,23,24,25,26]. Although serous, clear cell, and carcinosarcoma histologies represent only 10–15% of all EC cases, these minority subtypes account for up to 40% of all EC-related recurrences and subsequent deaths. This lower survival rate in comparison to endometrioid EC tumours is due, despite the fact that a growing list of evidence indicates the distinct nature of these subtypes at the molecular and clinical level, to non-endometrioid EC subtypes still being managed with the same adjuvant treatment as endometrioid EC tumours [27]. Similarly, the International Federation of Gynaecology and Obstetrics (FIGO) established a stratification system of EC according to its risk of recurrence based on postoperative pathologic information, such as histologic type, tumour grade, stage, and myometrial and lymphovascular invasion [26]. Nonetheless, 8% to 10% of early stage endometrial carcinoma develops recurrence and distant metastasis. Current classification systems have a limited potential to predict recurrence of EC patients, hence the need for more reliable systems to categorize and classify EC tumours to better tailor the clinical management of each individual patient.

Recently, the Cancer Genome Atlas Network (TCGA) performed an integrated genomic, transcriptomic, and proteomic analysis focusing on endometrioid EC and serous histologies, demonstrating that EC is a heterogenic disease. The TCGA identified and classified EC into four distinct molecular subgroups: POLE ultramutated (DNA polymerase epsilon), microsatellite instability hyper mutated, copy-number-low microsatellite stable, and copy-number-high serous-like [28]. Interestingly, TCGA molecular characterization data demonstrated that a quarter of the tumours classified by the dualistic model, as high-grade endometrioid EC, have a molecular phenotype similar to Type II serous non-endometrioid EC, including frequent TP53 mutations and extensive somatic copy number alterations (SCNA), thus suggesting that among these subtypes of patients similar clinical management should be considered beyond the histological classification. The molecular classification of EC has shown great promise, proving to be reproducible, demonstrating a higher association with clinical outcomes, and providing more valuable prognostic and predictive information in comparison to the dualistic classification system.

Considering that, researchers and clinicians are now enthusiastic and believe that a combination of both classification systems would be promising to precisely classify EC, thus improving the management of EC patients. However, the road to an ideal scenario should still be paved with a deeper understanding of the ability to predict treatment-response and with an increased variety of effective therapeutic options for the different types of EC. Moreover, inter and intra-tumour genetic heterogeneity represents a challenge that should be faced in the current classification systems as this might have implications for the response to standardized or personalized treatments [29]. In this sense, the development of clinical relevant models for translational research is of great importance, since they represent the disease and could translate the beneficial results of preclinical trial and drug screening assays into clinics to improve EC treatment therapy.

## 2. Endometrial Cancer PDX Models

### 2.1. Strategies for EC PDX Model Development

Even though PDX development is widely described [10,30], several steps may differ across different research groups regarding EC PDX development. In this review, which is co-authored by European Network of Individual Treatment in Endometrial Cancer (ENITEC) members, we analyze strategies for PDX model development and provide an overall perspective on the value of PDX models for EC research.

Cabrera et al. was the first to describe the development of orthotopic PDX models using human EC tumour tissue [31]. To develop this model, tumour tissue resected from an EC tumour was grown subcutaneously in nude mice previously to orthotopic implantation. Once the tumour engrafted subcutaneously, tumour was removed, mechanically crumbled, and injected transvaginally or transmyometrially into nude mice, generating the orthotopic PDX models. Between the two different methods for orthotopic PDX model generation, the transmyometrial implantation of the tumour had a higher engraftment rate compared to the tranvaginal injection. They showed that orthotopically implanted tumours produced myometrial infiltration, lymph-vascular invasion, and dissemination in the pelvic cavity. In addition, tumours retained the molecular and histological characteristics of the original samples, reproducing glandular patterns and expressing hormone receptors.

Similarly, Haldorsen et al. [32] reported the development of an orthotopic PDX by mechanical dissociation of a primary tumour biopsy into a cell suspension, which was injected into the left uterine horn of NSG mice. Unlike Cabrera et al., in which PDX models have to be euthanized after 63 days as a consequence of tumour invasion, presence of pelvic mass, and ascites development, Haldorsen et al. showed that their PDX developed from a cellular suspension took longer to reach this phenotype: almost 10 months. However, they showed that their orthotopically grown tumours could be excised, disaggregated into a cell suspension, and reinjected to develop a next-generation cohort of orthotopic PDX mouse models.

Later on, Depreeuw et al. [33] developed and fully characterized a panel of 24 subcutaneous EC PDX models that includes more frequent histologic and genetic subtypes of EC. The authors demonstrated that EC PDX models can be successfully established from both primary, metastatic, and recurrent endometrioid EC and non-endometrioid EC tumours, with an overall engraftment rate of 60%. They also showed that these models closely resemble the tissue architecture and genomic features of the original human tumours. By whole-exome sequencing focused on cancer consensus genes, they found that most of the mutations were common between the primary human tumour and its paired PDX model in four cases. In addition, they evaluated genomic copy number alteration in both samples and found that, on average, 90% of the genome had the same copy number alterations between the primary tumour and the xenograft. Similarly, by immunohistochemistry staining of PDX and a primary sample with a human vimentin antibody, they showed that human EC stroma is replaced by murine stroma after engraftment.

On the other hand, Unno et al. [34] established a xenograft model by transplanting fragments of four different EC histologies, from patients undergoing surgery, into the renal capsule of NSG mice. Following this approach, the authors reported that tumour xenografts retain characteristics of the original tumour and display features that are unique to endometrioid EC or non-endometrioid EC. In addition, they showed that each model has a different invasive and metastatic capacity according to its histology as well as a distinct dependence on β-estradiol [34]. Despite the technical difficulties presented by this type of approach, a renal capsule xenograft model is suitable for studying aggressive EC since tumour cells are in an environment susceptible to invasion and metastasis.

Recently, Pauli et al. [35] described the development of PDX from patient-derived tumour organoids (PDTOs). The authors collected metastatic and primary tumours from 18 different tumour types (two EC) and established culture organoids characterized by cytology and histology. Once established, PDTOs were subcutaneously injected into the flanks of nude mice. The xenograft take rate varied from 2 weeks to 16 weeks based on tumour type, and they reported an 86.4% engraftment rate of the PDTOs. They found that PDTOs and PDXs had similar histopathology to the parental tumours from which they were derived. The whole-exome sequencing genetic profile and single nucleotide variants analysis of PDTOs and PDXs showed excellent concordance with the patient’s tumour.

### 2.2. Strategies for EC PDX Model Monitoring

The ability to monitor disease development is one of the major challenges when using mouse models. Tumour xenografts growing subcutaneously could be followed simply by visual inspection or palpation; however, the monitoring of orthotopic tumour xenografts requires the utilization of imaging techniques to follow-up tumour progression. In this section, we will summarize the most commonly used in vivo imaging techniques that are currently available to monitor tumour growth in PDX models. A full description of these techniques is reviewed by Dall’Ara et al. [36].

- Micro-computed tomography is a high image resolution technique with great potential for in vivo use since it could be coupled with other imaging modalities providing three-dimensional (3D) reconstruction of bone and soft tissue. It has been used to study bone metastases [37] in mice and also has been applied to monitor the progression of lung and liver tumours [38,39,40].
- Magnetic resonance imaging (MRI) also offers in-vivo, non-invasive, 3D, and high-resolution images. In recent years, MRI has gained great importance due to the absence of ionizing radiation and its soft tissue contrast. Hence, MRI is used not only for its ability to define lesions with great spatial resolution but also to recover quantitative features that might be able to predict cancer progression.
- Positron emission tomography (PET) is a highly sensitive and specific imaging technique used to visualize the distribution and concentration of radiolabelled molecules injected into murine models. It is a form of quantitative whole-body imaging used for the in vivo monitoring of biological processes, such as enzymatic reactions, cellular metabolism, and cell proliferation and migration [41], which makes it an ideal tool for the imaging of cancer [32,42].
- Single-Photon Emission Computed Tomography is a technique that detects gamma radiation directly emitted by a radionuclide during decay and provides 3D information by acquiring multiple two-dimensional (2D) images while rotating around the imaged object. This technique is frequently used in oncology to visualize neuroendocrine tumours [43] and thyroid cancer [44,45] and to perform bone scintigraphy.
- Ultrasound is an ideal technique for detecting tumour growth in mice since it produces high-resolution images of small structures. It is a non-ionizing radiation technique, portable, easy to use, and quickly generates relevant images.
- Intravital microscopy is an optical imaging technique that enables highly sensitive in vivo imaging of tissue structure and function at high spatial resolution (cellular and sub-cellular) and temporal resolution. However, a surgical procedure is required to access the tissue/organ of interest for microscopy, having therefore the consequence that immediately after image acquisition the animal must be sacrificed. Intravital microscopy has been reported to be used in studies involving a metastatic process [46,47] and the response of tumour blood vessels to vascular targeted therapy [48,49].
- Whole-body optical imaging is a sensitive technique based on fluorochromes excitation by an external light source (fluorescence) or by chemiluminescent enzymatic light emission reactions within the animal (bioluminescence). Despite the poor spatial resolution due to light scattering, this technique enables the integration of the light signal emitted to obtain a 2D planar image. Green fluorescence protein (GFP) has been widely used to measure in vivo tumour growth as well as the effect of metastatic spread and drug treatments on different types of cancer in mouse models [50,51].

**Figure 1:**
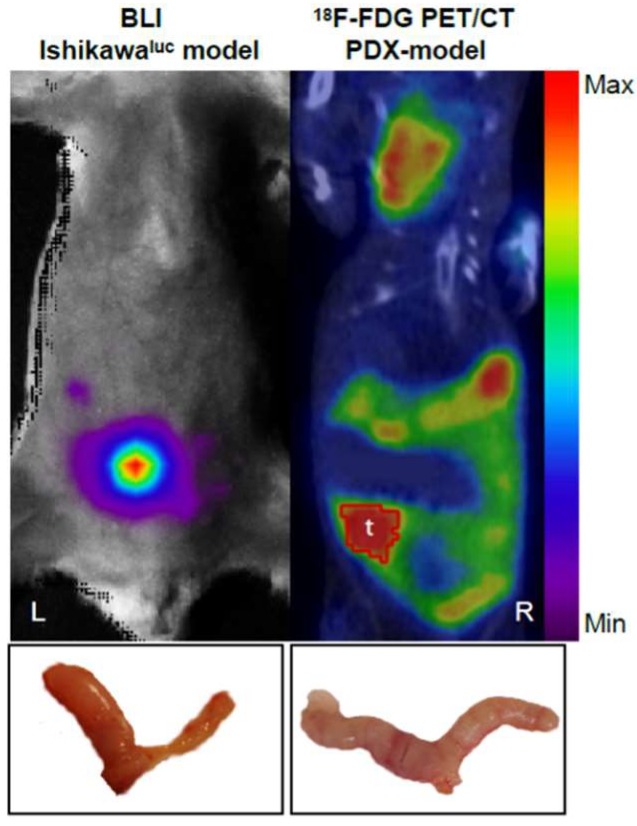
Comparison of imaging techniques for tumour evaluation. Bioluminescence imaging (BLI) enables the monitoring of cell-line-based orthotopic endometrial carcinoma, here demonstrated in a xenograft model generated from luciferase expressing Ishikawa cells (**L**). 18F-FDG PET/CT imaging is well suited for detection of endometrial carcinoma in PDX-models (**R**). Uterine tumours were confirmed by necropsy for each respective model (bottom).

Recently, Haldorsen et al. [32] described the use of a multimodal imaging platform based on a PET/CT scan and MRI in orthotopic EC cell-line-derived xenografts and PDX models and compared its relevance to bioluminescence imaging (BLI). In this study, they managed to monitor tumour growth, progression, and metastasis spread. Their findings bring forward the value of imaging techniques in the follow-up of orthothopic EC PDX models. Nevertheless, much research is needed to potentiate the use of imaging techniques for the follow-up of orthotopic EC PDX models. A comparative image of BLI and a PET/CT assay in mice models is shown in Figure 1.

### 2.3. Collaborative EC PDX Cohort

In a collaborative effort within the ENITEC consortium, we here describe in detail the PDX mouse cohort developed by the groups in Katholieke Universiteit (KU) Leuven, Haukeland University Hospital Bergen (HUHB), the Institute of biomedical research from Bellvitge–Institute Catalan of Oncology (IDIBELL-ICO), and the Vall d’Hebron Institute of Research (VHIR) in order to compile the information on EC PDX models available within this network. Altogether, we have generated a stable cohort of PDX models from primary tumour and metastasis from 124 EC patients recruited in different centres across Europe. This represents one of the most extensive and best-characterized EC PDX model cohorts available for research and is continuously increasing since we are generating new models from EC patients (Table 1).

We have developed a heterogenous population of PDX covering almost every stage and grade of both EC histological subtypes. In particular, we have 63 endometrioid and 56 non-endometrioid EC PDX models, in which serous and carcinosarcoma histologies are the best represented with 22 and 19 models, respectively. Regarding staging, 62% of the endometrioid EC PDX models are represented by FIGO stage I and classified according to Morice et al. [25] as low to intermediate risk of recurrence, while the rest of the endometrioid EC PDX models are represented by advanced stage III (21%) and stage IV (3%) tumours classified as high risk. Among the non-endometrioid EC PDX models, almost 60% are advanced tumours represented by FIGO stages III and IV, whilst 32% of the models are early stage tumours (Table 1). Analysing in depth our data, we observed that approximately the same number of PDX models were developed from both histologies, showing that endometrioid EC and non-endometrioid EC tumours could grow as PDX. Similarly, we do not observe a trend towards the specific growth of any EC tumour subtype (Table 2).

Our PDX cohort was developed both by heterotopic transplantation of fresh tumour tissue fragments recollected from a surgery room and implanted subcutaneously into athymic nude mice and by orthotopic implantation throughout a laparotomic incision. The engraftment rate of subcutaneous EC PDX varied from 60–80%; however, once the tumour was developed, the engraftment rate increased to nearly 100% in subsequent passages. In addition, PDX models take approximately 3–5 months to engraft and develop the first generation, while subsequent passages take less time to engraft and progress. In contrast, the orthotopic PDX model engraftment rate varies from 75–90% and also takes 2–5 months to develop a palpable and transferable tumour. Additionally, a small cohort of five tumours from EC patients was also injected orthotopically into the left uterine horn of NSG mice. In this case, a primary tumour sample is manually dissociated, filtered, and centrifugated and then a cell suspension is injected by laparotomy in a 1:1 proportion with Matrigel [32]. For this type of model, the engraftment rate is lower, ranging from 25 to 100% in the first generation, and the time of engraftment is also slower: it takes on average 10 months to develop an orthotopic EC PDX model.

**Figure 2:**
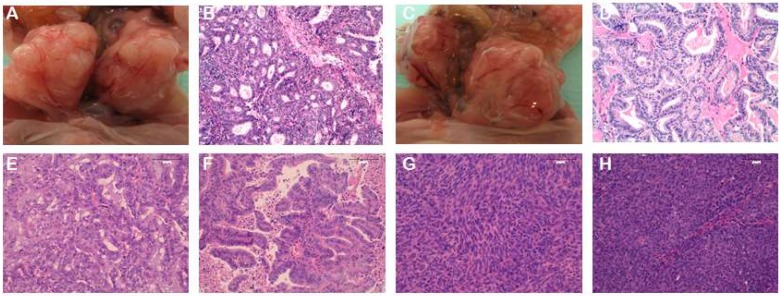
Representative PDX images from orthotopic (**A**–**D**) and heterotopic (**E**–**H**) models. (**A**–**D**) Orthotopic PDX from two different endometrioid EC patients. Panels A and C are a macroscopic image of the tumour growth in the uterus. Panels B and D represent images of the H&E staining of the PDX tumours. (**E**,**F**) H&E stainings from subcutaneous PDX models of two different endometrioid EC patients. (**G**,**H**) H&E stainings from subcutaneous PDX models of two different non-endometrioid EC patients. Panel G corresponds to a carcinosarcoma histology, and panel H corresponds to a serous carcinoma. Magnification 20×.

All of our models have been histologically characterized by H&E staining (Figure 2). Single-nucleotide polymorphism fingerprints analysis as well as the identification of gene mutations have been performed in some models. In the same way, we are working on the molecular characterization of PDX tumours by whole-exome sequencing and on the classification of those tumours according to the TCGA system. Finally, we have to mention that some of our PDX models have been used for preclinical drug-testing studies, described in the following section, with excellent results mimicking EC pathology and having a relevant response to treatment, demonstrating the high predictive value that PDX models could have in EC research.

### 2.4. Use of EC PDX Models in Preclinical Studies

Personalized medicine refers to the discipline focused on treating patients individually with molecular-targeted therapies directed against the altered pathways of their own tumour. This is expected to maximize treatment efficacy and minimize side effects [52]. Based on this notion, PDX provides a powerful tool for personalized medicine as it retains the molecular profile of the individual tumour. Similarly, many reports have shown that response rates in PDX correlate with those observed in the clinic both for targeted agents and for classic cytotoxic drugs [53,54]. Thus, the potential for using these models for directing individualized therapy in patients is being increasingly recognized [55].

In EC, the PI3K/AKT pathway is constitutively active due to mutations, and so this pathway has been an attractive target for therapy in different EC preclinical studies. Winder et al. tested the effect of MK2206, an allosteric inhibitor of AKT, on the growth and invasion of three EC PDX models grafted under the renal capsule of NSG mice. They found that MK2206 treatment inhibited tumour growth as well as decreased invasion into the kidney and spread throughout the peritoneum in the three different types of PDX (endometrioid EC grade 2, endometrioid EC grade 3, and non-endometrioid EC serous) [56]. Similarly, Yu et al. reported the use of two EC PDX models to study the effect of a two-drug combination, which acts on AKT (ARQ092) and FGFR1/2 (ARQ087), to overcome AKT inhibitor treatment loss of efficacy and resistance. FGFR is also frequently mutated in EC, promoting tumour progression and treatment resistance. Based on this, they suggested that by combining AKT and FGFR1/2 targeted therapy, they would be able to overcome the resistance mechanism. Even though they had a synergistic effect in EC cell lines, they only obtained enhanced antitumour activity in one of the PDX models compared to the single-agent treatment. However, the authors suggested that it is necessary to define EC patient molecular signatures, i.e., with mutations of PIK3CA/PIK3R1 and FGFR, to design a suitable treatment strategy and predict patients’ response [57].

The use of palbociclib against the cyclin-dependent kinases CDK4 and CDK6 has successfully been used in advanced breast cancer [58] and it is under evaluation in many other cancer types in phase II and III clinical trials [59]. In EC, Dosil et al. [60] performed the first preclinical study to test the therapeutic potential of palbociclib; specifically, this was tested in the endometrial malignancies driven by *Pten* deficiency. This work started with the assessment of palbociclib response in vitro and in a PTEN-deficient GEM model and was finally validated in a PTEN-mutated PDX model of endometrioid EC of FIGO stage IIIC and grade 2. This work evidenced that palbociclib has therapeutic potential as an anticancer drug in the endometrium, since it reduces tumour cell proliferation and disrupts the tumourigenesis process [60].

Similarly, Dupreeuw et al. [33] tested the efficacy of NVP-BEZ235 (a dual pan-PI3K/mTOR inhibitor) and AZD6244 (an MEK1/2 inhibitor) in a PDX model harbouring a high-grade recurrent endometrioid carcinoma carrying PTEN, PIK3CA, and KRAS mutations. They showed that the treatment as a single therapy significantly reduced tumour growth compared to the control group. Moreover, when combining both therapies, NVP-BEZ235 and AZD6244, the treatment was as effective as Carboplatin, resulting in disease stabilization showing no increment of tumour growth.

All of the above-mentioned studies shared a similar approach to evaluating the efficacy of targeted therapies in EC: all of them relied on the use of PDX to validate a specific treatment which was first assessed in one or more in vitro and/or in vivo models. Moreover, PDX models could also be used to identify pathways responsible for therapy-resistant mechanisms and to identify new approaches to overcome any acquired resistance in EC tumours. Sorafenib, an antiangiogenic drug, has been proposed as a promising targeted therapy for EC, but a multicentre phase II clinical trial demonstrated moderate effects. In a recent work, Eritja et al. [61] studied the resistance mechanism of sorafenib in EC and demonstrated that autophagy acted as a protective mechanism against sorafenib. They developed in vitro assays and three different endometrial orthotopic xenografts (endometrioid EC grade 1, 2, and 3), and observed that the inhibition of autophagy by using cloroquine potentiates sorafenib effects in PDX orthotopic EC tumours. These results provided insights into the modest effects of sorafenib trials in EC patients and might open new avenues for the design of preclinical studies using sorafenib.

Equally important is the discovery of biomarkers to predict treatment-response, as this will help to tailor the treatment of EC patients. In this field, Groeneweg et al. [62] investigated the effectiveness of *HER2* inhibition in serous non-endometrioid EC. The combination of in vitro and in vivo cell-line and PDX models permitted the authors to demonstrate that lapatinib as a single agent and in combination with trastuzumab induced significant tumourstatic effects only in those tumours harboring *HER2* gene amplification. In the non-amplified tumour xenografts, a complete lack of response to any administered therapy was seen. Thus, this study unveiled that *HER2* gene amplification might be used a biomarker for response to *HER2* inhibition in uterine serous carcinoma, as has been shown in breast and gastric carcinomas. Similarly, in another study published by Groeneweg et al. [63], they demonstrated that the expression of nuclear Notch1 could be associated with tumour progression since it was expressed in a significant proportion of endometrioid ECs (12%) as well as in the majority of serous non-endometrioid EC analysed (58%). They showed that treatment with the gamma-secretase inhibitor MRK-003 decreased the proliferation of serous cell lines in vitro and restricted the growth of xenografts derived from serous cell lines and primary human serous tissue in vivo. Moreover, MRK-003 treatment augmented the anti-tumour activity of standard Paclitaxel/Carboplatin (P/C) therapy in one of the two primary human PDX models. The observed synergistic effect of MRK-003 with conventional P/C therapy in one primary model provides pilot data to suggest that the combination of a Notch inhibitor and standard chemotherapy may have promise in the management of serous carcinoma.

Based on these data, EC PDX models are currently playing an important role in defining new therapeutic options for the different EC subtypes and helping in the selection of populations of patients most likely to be sensitive to a new agent. However, all the studies performed up to date only include from a single to a few EC PDX models; thus, the results are hard to translate into larger populations.

## 3. New Perspectives on the Use of EC PDX Models

There exist interesting avenues for the exploitation of PDX models, which have been explored in other types of cancers but not in the field of EC research. A co-clinical trial is a concept, similar to personalized PDX models, which is based on the development of a PDX from a patient enrolled in a clinical trial and treated in the same way as the patient [30,64]. This strategy provides an interesting platform for the identification of predictive or response biomarkers and on which to assess the therapeutical benefit of novel combinations. Limitations of co-clinical trials include the limited ratio of engraftment of PDX models and the extended time for PDX development of particular cases, which might impair the evaluation of the PDX response for all patients recruited in the clinical trial. Moreover, PDX models do not always faithfully represent primary tumour heterogeneity and, in these cases, PDX therapy response would not be relevant for the patient.

Another interesting approach is the use of PDX panels for preclinical studies as opposed to the traditional methods of assessing drugs in just a few models [65,66]. This approach uses PDX models as if those were patients participating in a phase II clinical trial, i.e., a cohort of different PDX models covering different types and stages of cancer are used, and only one or a few animals with specific characteristics are included per patient and receive a specific treatment. When analysing the responses to treatments, it is not the response of an individual mouse/tumour that matters, but the population response. It should be noted, however, that to completely capture the full inter-tumour heterogeneity of a particular cancer type, large panels of PDX are needed to cover different subtypes, stages, and grades of differentiation. Considering this, we have to mention that this approach is not feasible for tumour types that poorly establish as xenograft models.

Migliardi et al. [65] was the first to describe this mouse clinical trial approach. Later on, Gao et al. [67] proposed the use of just one tumour representing “a patient” to allow for even greater efficiency (the 1 × 1 × 1 approach), determining that using just the one animal per cohort study design has outstanding reproducibility for the data collected. The utility of this approach is that it enables many more types of PDX and treatment groups to be assessed operationally, and the inter-heterogeneity of patients can be captured experimentally. This approach is closely related to a clinical study in patients and is being used by some researchers and pharmaceutical companies motivated to increase the success rate of drugs tested in preclinical phases that are finally approved by regulatory agencies.

## 4. Collaborative PDX Networks

At present, the cost and resources needed for PDX development and maintenance are a limiting factor for many researchers in order to develop their own models. For this reason, to facilitate working with PDX models, accessible collaborative networks between academic research groups have been established that closely cooperate with the clinic in order to associate preclinical experimentation and clinical activities. Such networks include the EurOPDX Consortium, the U.S. National Cancer Institute (NCI) repository of patient-derived models, the U.S. Pediatric Preclinical Testing Consortium (PPTC), the Children’s Oncology Group (COG) cell culture and xenograft repository, the Public Repository of Xenografts (PRoXe), and the Novartis Institutes for Biomedical Research PDX Encyclopedia (NIBR PDXE) [68]. Some of the PDX models developed in EC in the ENITEC consortium are included in the EurOPDX network.

Besides academic collaborations, commercial companies have also started to provide PDX models, such as The Jackson Laboratory (Bar Harbor, ME, USA), Xenopat (Barcelona, Spain), CrownBio (Santa Clara, CA, USA), Oncotest GmbH (Freiburg, Germany), AVEO Oncology (Cambridge, MA, USA), Living Tumour Laboratory (Vancouver, BC, Canada), Urolead (Strasbourg, France), Experimental Pharmacology & Oncology Berlin-Buch GmbH (Berlin-Buch, Germany), and XenTech (Paris, France) [69].

## 5. PDX-Related Challenges

Although EC PDX models have been established for almost a decade, there are still several challenges that should be faced in the near future. First, immunocompromised mice are used so as to not reject human tumour, meaning that immunologically related aspects cannot be taken into account. Next, human stroma in mice is replaced by murine stroma over different generations. This has been shown for different types of tumours, but also more specifically for EC [33]. Both immunological and stromal cells are part of the tumour microenvironment, and it is known that they are implicated in cancer progression and metastasis [70]. Therefore, there is a need to investigate the tumour microenvironment [71]. Third, although tumour heterogeneity is maintained, only small pieces of primary tumours are used, which means there is potential for loss of tumour information. Indeed, efforts to reproduce clear intra-tumour heterogeneity in different murine models should be addressed. Finally, it is becoming clear that, although the pharmacological and most of the original biological characteristics are maintained, tumours partially undergo mouse-specific evolution. More specifically, Ben-David et al. monitored the SCNAs of 1110 PDX models for 24 cancer types over time. They did find an accumulation of SCNAs over time that correlate with the primary tumour. However, several SCNAs observed in primary tumours disappear in PDX models and SCNAs acquired by PDXs differ from primary tumours [72]. Other studies suggest that, although there is indeed engraftment-associated selection, the majority of changes do not occur in oncogenic drivers and are therefore not affecting intra-tumour heterogeneity [33,68,73].

To overcome the lack of tumour microenvironment and immune cell interaction, humanized mice can be used [74]. Humanized models can be used to investigate cancer stem cells amongst other biological facets, such as tumour–microenvironment interactions and anti-tumour immune responses, and they can be used in immunotherapy research [75]. For endometrial cancer, no reports using humanized mice have been published yet. However, humanized mice have been successfully established for haematological malignancies [75] and many types of cancer [74,75]. Different methodologies can be used to generate humanized models; however, this subject exceeds the scope of this article and excellent reviews about humanized models can be found in the literature [68,73,75].

## 6. Conclusions and Future Perspectives

Over the last few decades, there has been increasing interest in developing more realistic and clinically relevant mouse models. In this context, PDX emerges as a promising preclinical mouse model mainly because it faithfully retains patient tumour characteristics and behaviour. Importantly, EC PDX models have already been used in an individualized approach to evaluate the efficacy of novel therapies and to identify biomarkers to predict treatment-response. Together with the advances of omics techniques, which allow us to increase our understanding of the molecular alterations of EC tumours and dysregulated EC pathways, EC PDX models are now an untapped source to improve the definition, consecution, and output of preclinical studies to increase the success ratio in further clinical phases. In this review, we compiled the information on EC PDX models that have been described in the literature and highlight the models that have been generated in the ENITEC consortium, from their generation to their use, and identify new perspectives and limitations of those models.

## Figures and Tables

**Table 1:** PDX models developed by European Network of Individual Treatment in Endometrial Cancer (ENITEC) consortium members.

Research Centre	Type of Sample	Type of PDX	Tissue	Engraftment Rate & Time		Mouse Strain	Number Models	Type of Models	Preclinical Drug Tested
IDIBELL-ICO	Primary tumor, metastases	orthotopic	small tissue fragment	75–90%	1–5 months	Athymic nude	64	60%EEC; 10%PS; 20%CS; 3%CC; 7%other types	Sorafenib, Chloroquine (61)
VHIR	Primary tumor, metastases, recurrences	heterotopic (s.c)	5–10 mm^3^ tissue fragment	60–80%	2–3 months	Athymic nude	40	43%EEC; 32%PS; 10%CS; 2.5%CC; 5%undifferentiated 7.5%other types	Carboplatin Paclitaxel, Palbociclib (60)
KUL	Primary tumor, metastases, recurrences	heterotopic (s.c)	8–10 mm^3^ tissue fragment	100%	3–5 months	Athymic nude	15	46%EEC; 13%PS; 13%CS; 7%undifferentiated 21%other types	Carboplatin, NVP-BEZ235, AZD 6244 (33)
HUHB	Primary tumor, metastases	orthotopic	Cell suspension	25–100%	3–13 months	NSG	5	60%EEC; 20%PS; 20%undifferentiated	

IDIBELL-ICO: Institute of biomedical research from Bellvitge–Institute Catalan of Oncology; VHIR: Vall d’Hebron Institute of Research; KUL: Katholieke Universiteit Leuven; HUHB: Haukeland University Hospital; EEC: Endometrioid endometrial cancer; PS: Papillary serous carcinoma; CS: Carcinosarcoma; CC: Clear cell carcinoma; s.c: Subcutaneous.

**Table 2:** ENITEC PDX models classified according to histology, stage, and differentiation grade.

		Endometrioid EC		Non-Endometrioid EC	
FIGO stage	I	39	62%	18	32%
	II	7	11%	4	7%
	III	13	21%	27	48%
	IV	2	3%	5	9%
Grade	1	20	32%		
	2	23	37%		
	3	20	32%	56	100%
Histology	Serous carcinoma			22	39%
	Carcinosarcoma			19	34%
	Clear Cell carcinoma			4	7%
	Others			11	20%

## Abbreviations

GEM	Genetically engineered mouse
PDX	Patient-derived xenograft
SCID	Severe combined immunodeficient mice
NOD	Non-obese diabetic
NSG	NOD/SCID gamma mice
FIGO	International Federation of Gynaecology and Obstetrics
EC	Endometrial cancer
TCGA	The Cancer Genome Atlas
SCNA	Somatic copy number alterations
POLE	DNA polymerase epsilon
ENITEC	European Network of Individual Treatment in Endometrial Cancer
PDTO	Patient-derived tumour organoids
CT	Computed tomography
MRI	Magnetic resonance imaging
PET	Positron emission tomography
GFP	Green fluorescence protein
BLI	Bioluminescence imaging
IDIBELL-ICO	Institute of biomedical research from Bellvitge–institute Catalan of Oncology
VHIR	Vall d’Hebron Institute of research
H&E	Hematoxylin and eosin
AKT	Alpha serine threonine kinase
FGFR	Fibroblast growth factor receptor
CDK4	Cyclin dependent kinase 4
CDK6	Cyclin dependent kinase 4
PTEN	Phosphatase and Tensin Homolog
NCI	National Cancer Institute
PPTC	Pediatric Preclinical Testing Consortium
COG	Children’s Oncology Group
PRoXe	Public Repository of Xenografts
NIBR PDXE	Novartis Institutes for Biomedical Research PDX Encyclopedia
